# Exposure to work-related violence and/or threats of violence as a predictor of certified sickness absence due to mental disorders: a prospective cohort study of 16,339 Swedish men and women in paid work

**DOI:** 10.1007/s00420-022-01917-w

**Published:** 2022-09-07

**Authors:** Maria Wijkander, Kristin Farrants, Linda L. Magnusson Hanson

**Affiliations:** 1 Stress Research Institute, Department of Psychology, Stockholm University, 10691 Stockholm, Sweden; 2 Division of Insurance Medicine, Department of Clinical Neuroscience, Karolinska Institute, Stockholm, Sweden

**Keywords:** Work-related violence, Work-related threats, Stress, Sickness absence, Mental disorder

## Abstract

**Objectives:**

The aim of this prospective cohort study was to investigate if exposure to work-related violence and/or threats of violence predict certified sickness absence due to mental disorders.

**Methods:**

Information on work-related exposure to violence and/or threats of violence were derived from the biannual Swedish Longitudinal Occupational Survey of Health (SLOSH) study 2012–2016, including individuals in paid work across Sweden and from different occupations/sectors (*n* = 16,339). Certified sickness absence due to mental disorders were ascertained from register data from the Swedish Social Insurance Agency. Odds ratios of certified sickness absence due to mental disorders according to exposure to work-related violence were estimated using multiple logistic regression. Several potential confounding variables, such as demographic and socio-economic factors, age, sex, cohabitation, children living at home, socio-economic status, educational level, as well as other types of psychosocial work environmental factors, were adjusted for in the analyses.

**Results:**

In the total study sample, 9% reported exposure to violence and/or threats of violence and the prevalence of sickness absence due to mental disorders was 5%. Exposure to work-related violence and/or threats of violence was associated prospectively with certified sickness absence due to mental disorders (odds ratio 1.46, 95% confidence interval 1.17–1.82, *p* < *0.01*). Analysis of possible interaction showed no difference in association when comparing women to men and different age groups.

**Conclusions:**

Exposure to work-related violence and/or threats of violence appear to increase the odds of certified sickness absence due to mental disorders. Preventive measures aiming to lower the risk of exposure is thus of great importance.

**Supplementary Information:**

The online version contains supplementary material available at 10.1007/s00420-022-01917-w.

## Background

Long-term sickness absence, in Sweden, usually defined as more than 60 days’ absence from work that the employer accepts being attributable to sickness (Lidwall 2015), has significant societal costs in terms of production losses and health-care costs, as well as potential major suffering for the individual due to lowered quality of life, isolation and exclusion from certain aspects of society (Henderson et al. 2011; Lidwall et al. 2018). While a temporary decrease in numbers of certified sickness absence in Sweden was observed during the early twenty-first century, the level began to rise again around year 2010 (Lidwall et al. 2018). A similar trend has been observed for several European countries, including Germany, the United Kingdom and the Netherlands, as well as the other Scandinavian countries Norway and Denmark, where there has been an increase in proportion of people on sickness absence (Henderson et al. 2011; Krane et al. 2014; Lund et al. 2009; Markussen and Røgeberg 2012; Vaez et al. 2007). Especially, sickness absences related to mental disorders (disorders associated with clinically significant disturbance in cognition, emotional regulation, or behavior) have increased in Sweden recently (Lidwall 2020). This includes disorders such as depression, anxiety disorders, and stress-related disorders (e.g. diagnosis of chronic exhaustion disorder, a diagnosis to date uniquely used in Sweden (Socialstyrelsen 2003)), often referred to as common mental disorders (Kendrick and Pilling 2012). These types of disorders have also become the main cause of sickness absence among most OECD (Organization for Economic Co-operation and Development) countries (*Mental Health and Work*, OECD 2013; *Sick on the Job?*, OECD 2012) and are typically longer than sickness absences due to other diagnoses (Henderson et al. 2011; van Hoffen et al. 2020). Furthermore, sickness absence due to common mental disorders has a high rate of recurrence, with prior studies showing that 65% of employees with sickness absence due to common mental disorders also had high levels of sickness absence in the following years (Koopmans et al. 2011). Sickness absences due to common mental disorders are therefore associated with an exceptional high social and economic burden.

There are also some indications of an increase in violence and harassment over time in Europe, especially violence originating from a third part (for example from customers, clients, patients and students) (*Violence and Harassment in European Workplaces*, Eurofound 2015). Workplace violence is according to the ILO (International Labor Organization) defined as “any action, incident or behavior that departures from reasonable conduct in which a person is assaulted, threatened, harmed or injured in the course of, or as a direct result of, his or her work” (*Work-Related Violence*, ILO 2015, p.11) and can include both physical assaults such as beating, and psychological assaults, such as threats and harassments. Work-related violence has been reported to be particularly prevalent in occupations or sectors such as health care, social work, transportation and storage, accommodation and food services, public administration as well as education (*Violence and Harassment in European Workplaces*, Eurofound 2015). Some previous studies, for example, show that 33% of all men and 31% of all women employed in health care or social work in Sweden report being exposed to such negative social behavior during the last period of 12 months (Nyberg et al. 2020) and over 61% of all health care workers globally are estimated to be exposed to workplace violence (Liu et al. 2019).

Earlier studies have suggested that exposure to work-related violence and/or threats of violence is associated with an increased risk of different types of negative mental health consequences such as depression, fatigue and nervousness (Rudkjoebing et al. 2020, 2021). Additionally, several previous studies have suggested that there is an association between exposure to work-related violence and/or threats of violence and sickness absence (Aagestad et al. 2016; Friis et al. 2018; Niedhammer et al. 2013; Nyberg et al. 2021; Slany et al. 2014; Sterud et al. 2021). However, many previous studies focusing on exposure to violence and/or threats of violence and sickness absence have limitations. Several studies rely completely on self-reported data (Andersen et al. 2019; Magnavita 2013; Niedhammer et al. 2013; Sterud et al. 2021) for both exposure and outcome variables, which could mean a risk for common method bias (variance owing to the measurement method rather than the constructs of interest potentially caused by e.g. mood state, social desirability, predictor and criterion variables measured using the same medium) (Podsakoff et al. 2003). Furthermore, several studies focus on one particular occupation (e.g. employees in the health care sector) (Lanctôt & Guay 2014; Li et al. 2020; Mento et al. 2020) and also base their analyses on fairly small study samples, which could make it difficult to draw valid conclusions from the results and limits the possibility to generalize to the population as a whole. Most evidence concerns psychological violence (bullying and/or harassment) rather than physical violence (Nyberg et al. 2021). Furthermore, to our knowledge only a few previous studies focusing on the consequences of exposure to workplace violence and/or threats of violence have used register data of certified sickness absence as an outcome variable, none of which specifically examined sickness absence due to mental disorders (Hoffmann et al. 2020; Sterud et al. 2021). Thus, there is a lack of studies investigating the potential association between workplace violence and sickness absence due to diagnosed mental disorders.

The present study addresses the need for relatively large and representative studies with independent measures of exposure and outcome and a valid measurement of sickness absence specifically due to diagnosed mental disorders. The overall aim of the present study was to investigate if exposure to work-related violence and/or threats of violence predict later certified sickness absence due to mental disorders.

## Methods

### Study sample and data

#### Swedish Longitudinal Occupational Survey of Health (SLOSH)

SLOSH is a large prospective cohort study aiming to investigate longitudinal associations between work organization, work environment, labor force participation, health and well-being, taking social conditions, individual differences, health behaviors, coping strategies, work-private life interaction, sleep and ageing into account. The SLOSH cohort currently consists of 40,877 participants in the Swedish Work Environment Surveys 2003–2011 (conducted every second year), successively included in the total cohort (Magnusson Hanson et al. 2018). These participants were originally recruited by stratified (county, sex, citizenship, inferred employment status) random sampling from the entire country to the Labor Force Survey (LFS). A sample of working individuals aged 16–64 years was then selected from LFS for the Swedish Work Environment Survey (SWES). SLOSH is thereby based on a nationally representative sample of the working individuals from all occupations/sectors and parts of the country. The cohort is followed-up via official registers on, e.g. certain demographic factors, occupational factors, benefits and hospital care. In addition, self-reported data have been collected biannually since 2006, on a successively larger part of the cohort over time (Magnusson Hanson et al. 2018). Data from questionnaires and registers are linked via the Swedish personal identification numbers, replaced by serial numbers (pseudonymized data) for the study.

At each data collection, the participants are asked to answer one of two self-reported survey questionnaires depending on whether they are in paid work for ≥ 30% of full-time or whether they are working less than 30% or not working at all at the time the questionnaire was answered. If primarily in paid work, the participants respond to a range of questions regarding organizational and psychosocial work characteristics, social factors, and indicators of health and wellbeing. The questionnaire that was directed towards individuals currently not in paid work > 30% instead focuses on factors related to having left active working life temporarily or permanently in addition to questions regarding health and social factors.

For this prospective cohort study, data from three waves (2012–2016) of SLOSH were used. The response rate for the three waves of survey included in the study was just above 50%. In 2012, 9880 individuals aged between 16 and 64 years; 7325 responded to the questionnaire directed to “workers” , i.e. individuals in paid work for ≥ 30% of full-time, and 2555 answered the questionnaire directed to “non-workers, i.e. individuals working less than 30% of full time or not working at all. In 2014, the number of invited individuals was larger and 20,316 responded to the survey; 15,359 responded to the “worker questionnaire” and 4957 responded to the “non-worker questionnaire”. In the third wave, administered in 2016, 19,360 responded: 13,572 to the “worker questionnaire” and 5788 to the “non-worker questionnaire”.

**Fig. 1:**
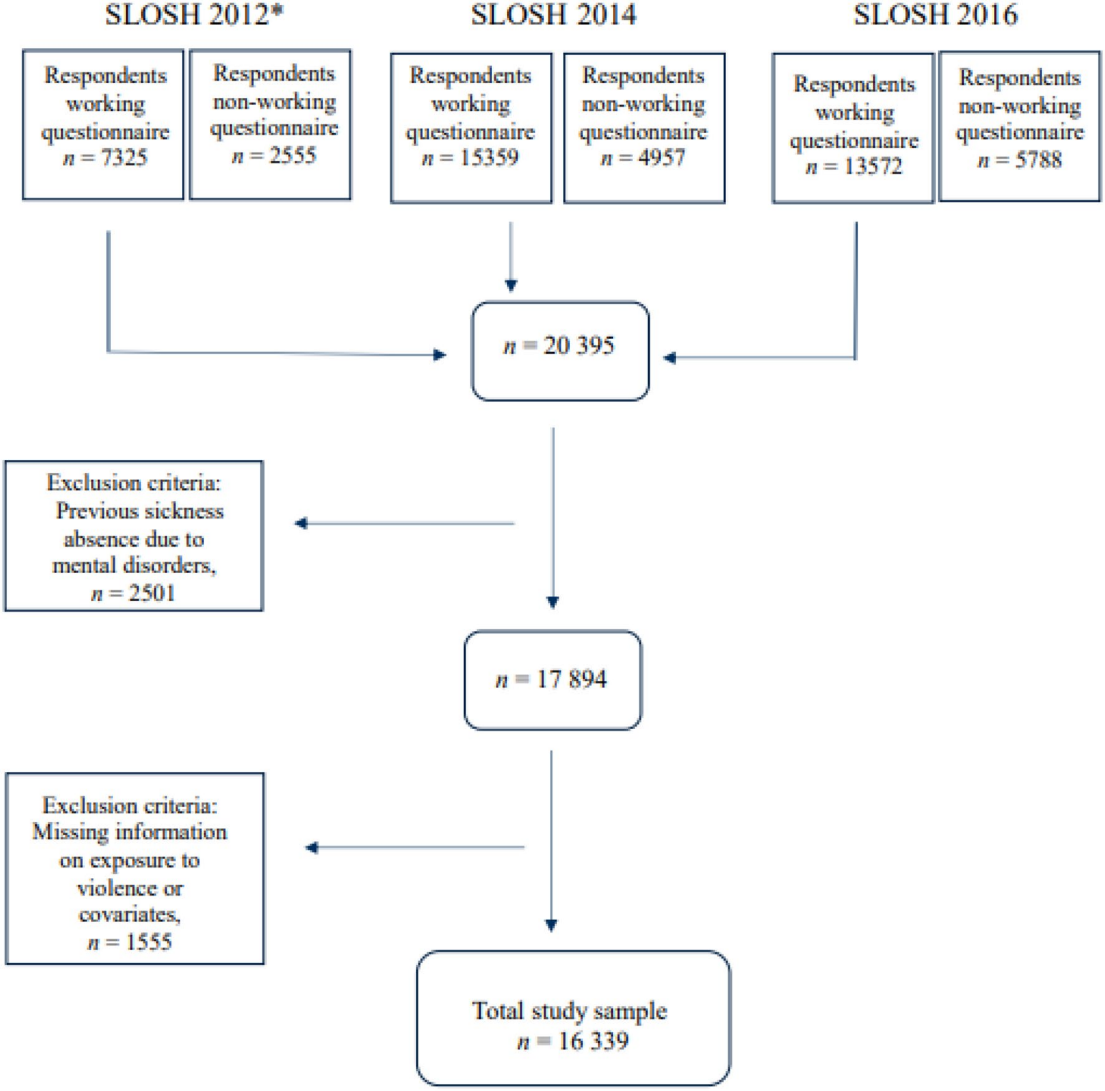
Selection of study samples. “Working questionnaire” for those respondents working ≥ 30% of full time. “Non-working questionnaire” for those respondents working ≤ 30% of full time or not working at all. * In SLOSH 2012 only participants from SWES 2003 and 2005 were contacted (*n* = 18,917) while for SLOSH 2014 and 2016, participants from SWES 2003, 2005, 2007, 2009 and 2011 were invited (*n* = 40,877)

The final study sample (*n* = 16,339) consisted of respondents to the survey directed to those in paid work for ≥ 30 of full time either 2012 (*n* = 7325), 2014 (*n* = 15,359) or 2016 (*n* = 13,572), who also had valid information on work-related violence, and the selected covariates. If the participants had responded in 2012, 2012 was considered the baseline for this study. Otherwise, 2014 or 2016, whichever was the first wave of participation, was considered baseline for this study. Individuals with previous sickness absence due to any mental disorder up to baseline were also excluded from the final study sample, see Fig. 1.

### Variables

#### Exposure

Exposure to violence and/or threats of violence was assessed at baseline with a question asking whether they, during the past 6 months, had been exposed to violence and/or threats of violence in their job. Four different response options were possible: “*Yes, one or several times/week*”, “*Yes, one or several times/month*”, “*Yes, sometime during the last 6 months*”, “*No*”*.* The respondents were then categorized into two groups based in their answers: “*Yes*” (= 1) or “*No*” (= 0), where the first three options were all interpreted as “*Yes*”.

Additionally, to investigate whether there is a dose–response relationship between the exposure and outcome, exposure to violence and/or threats of violence was divided into three

different categories; *0* = *no exposure*, *1* = *occasional exposure—sometime during the past 6 month or one or several times/months*, *2* = *frequent exposure—one or several times/week.*

#### Outcome

Doctor certified sickness absence due to a mental disorder (defined according to the International Classification of Diseases version 10, ICD-10 diagnosis within chapter V F00-F99) was used as outcome variable in the present study. Data on certified sickness absence was retrieved from the MiDAS database (Micro Data for Analysis of Social Insurance), which is a database held by the Swedish Social Insurance Agency (SSIA) covering year 1994 to 2018. MiDAS contain information on payments of all types of sickness- and rehabilitation benefit payments as well as diagnoses and durations of sickness benefit spells in Sweden. Participants were followed from baseline until 2018 resulting in a follow-up period of up to 6 years for participants with baseline 2012 and a maximum of 2 years for participants with 2016 as baseline. All spells of sickness benefit as well as rehabilitation benefit with a duration of 14 days or longer was included in the analysis. Diagnoses of mental disorders included in the study were among others, major depression, Post-traumatic stress disorder (PTSD), Acute Stress disorder (ASD) and exhaustion disorder.

#### Covariates/confounding

From the results of previous studies in the same research field a number of variables were identified as possible confounders (Heming et al. 2021; Leineweber et al. 2019, 2020; Rudkjoebing et al. 2020). Among these, age, sex, cohabitation, children living at home, educational level and socio-economic status were considered as well as other work-environmental factors such as support from supervisors and co-workers, demands and decision authority. Information on educational level was retrieved from a database held by the Statistics Sweden (SCB) and was divided into four different categories: maximum of nine years of compulsory education, upper secondary education, university < 3 years and university ≥ 3 years including post graduate education.

Socio-economic status was coded using a classification system consisting of five different socio-economic groups: group one included unskilled employees in goods or service production with a normal educational level of < 2 years following compulsory and upper secondary education, group two consisted of skilled employees in goods or service production with an educational level normally of > 2 years, group three consisted of assistant non-manual employees with an educational level normally of < 3 years. Group four consisted of intermediate non-manual employees with a normal educational level of 3–5 years and the last group (group five) consisted of professionals and other high non-manual employees, including upper-level executives, with a normal educational level of at least six years of education following compulsory and upper secondary education.

Information on age and sex was obtained from register data through personal identification numbers, while information on cohabitation, children living at home as well as information on psychosocial work environmental factors (support, demands and decision authority) was obtained through SLOSH. Cohabitation and children living at home were analyzed as dichotomous variables where participants either were considered to be living with a spouse (married or cohabiting) or not, and either having children living at home (for at least 50% of time) or not. The psychosocial work environmental factors support, demands and decision authority, were measured using several items in the SLOSH-questionnaire where answers were given on four-point Likert-scales. Support was measured using six items regarding whether the participant thought there was a good atmosphere at the workplace, within the work group and between employee and superior, such as “*There is a good spirit of unity*”, “*My colleagues are there for me*” and “*I get on well with my superiors*”*.* To measure level of demands the participants were requested to answer five questions regarding the work such as “*Do you have to work very fast?*”, “*Do you have to work very intensively?*” and “*Does your work demand too much effort?*”. Decision authority was measured using two questions asking if the participant had authority to decide *what* and *how* to do the work i.e. “*Do you have a choice in deciding what you do at work?*” and “*Do you have a choice in deciding how you do your work?*”. Each set of items used to measure the different psychosocial work environment factors was computed into an index providing mean levels for each respective measurement, where high values indicate high demands, low social support and low decision authority. These indices were subsequently handled as continuous variables in the analysis. To investigate if psychosocial work environment factors interact with work-related violence, each measure was treated as a dichotomous variable, i.e. social support was divided into *high* and *low social support*, demands was divided into *high* and *low level of demands* and decision authority was divided into having *high* or *low decision authority* according to the median levels of *support, demands* or *decision authority*.

### Statistical analysis

Statistical analyses on the data were performed using IBM SPSS 27.

Crude and adjusted odds ratios (OR) and 95% confidence intervals (CI) calculated by means of multiple logistic regression analyses were used to estimate the odds of sickness absence among workers who experienced exposure to work-related violence and/or threats of violence at baseline. In the crude model, only exposure to violence/and or threats of violence was included as a predictor in the model. In model 1 the model was adjusted for possible confounding variables such as demographic factors such as age, cohabitation, children living at home, educational level and socio-economic status. Model 2, which was considered the fully adjusted model, was further adjusted for psychosocial work environmental factors; support, demands and decision authority.

The crude model, model 1 as well as model 2 were all further analyzed using the exposure variable in the form of categorical variable to be able to elucidate a possible dose–response relationship.

A possible interaction between sex and exposure to violence and/or threats of violence was also tested in a complementary analysis, by including an interaction term in the regression model adjusting for possible confounding factors such as age, sex, cohabitation, children living at home, socio-economic status, educational level and other work environment factors. In addition, we tested for interaction between violence and/or threats of violence and age. In these analyses age was categorized into the following groups: the first group included individuals ≤ 35 years of age, the second group ages 36–55 and the third group consisting of individuals ≥ 56 years of age. Possible interactions were also tested between work-related violence and/or threats of violence and social support at work, work demands and decision authority.

## Results

### Descriptive statistics

Table 1 presents descriptive statistics for the study sample. A higher proportion of the participants in the study were women (54%) than men and the sample had a mean age of 50 years. A predominant part of the sample (81%) was living with a partner in marriage or cohabitation. Just above half of the sample reported having children living at home (52%). Most of the participants had attainted university level education (51%). As much as 43% reported having a university education of more than 3 years. Almost a third of the sample were intermediate non-manual employees working in positions which normally require an educational level of 3–5 years following compulsory and upper secondary education (32%).

**Table 1:** Baseline characteristics of the study sample

Characteristic	Total		Exposed to workplace violence and/or threats of violence *n* = 1433 (9%)		Not exposed to work-place violence and/or threats of violence *n* = 14 906 (91%)	
	*n*	%^a^	*n*	%^a^	*n*	%^a^
Sex						
Women	8772	54	1013	70	7759	52
Men	7567	46	420	30	7147	48
Age						
Mean age (SD)	49.74 (10.3)		48.43 (10.4)		49.86 (10.3)	
Cohabitation						
Married/cohabiting	13,172	81	1120	78	12 052	81
Living alone	3167	19	313	22	2854	19
Children living at home						
Yes	7855	48	696	49	7159	48
No	8484	52	737	51	7747	52
Educational level						
Compulsory education (max 9 years)	1118	7	59	4	1059	7
Upper secondary education	6952	43	639	45	6313	42
University < 3 years	1213	7	68	5	1145	8
University > 3 years	7056	43	667	47	6389	43
Socio-economic groups						
Group 1, unskilled manual workers	2432	15	217	15	2215	15
Group 2, skilled manual workers	2658	16	417	29	2241	15
Group 3, assistant non-manual workers	2238	14	112	8	2126	14
Group 4, intermediate non-manual workers	5283	32	514	36	4769	32
Group 5 professionals and other higher non-manual workers	3728	23	173	12	3555	24

^a^ Of total

For the entire study sample, the prevalence of exposure to workplace violence and/or threats of violence was 9%.

Just below 5% of the total sample had had one or more spells of sickness absence due to mental disorders after baseline (2012, 2014 alternatively 2016).

Of all of those participants who had a new sickness absence spell due to mental disorder after baseline, 15% reported being exposed to work-related violence and/or threats of violence during the past 6 months.

In our study sample a higher proportion of women reported exposure to work-related violence compared to men (12% and 6%, respectively). Likewise, the proportion with sickness absence among women was also higher compared to among men (7% vs 2%, respectively).

When stratifying the study sample by age, it was found that the group consisting of the youngest participants, ≤ 35 years of age, had the highest prevalence of exposure to work-related violence and/or threats of violence (11%), followed by the next age category (36–55 years of age) with a prevalence of exposure of 9.2%. Among the group consisting of participants of < 35 years of age and the group of participants 36–55 years of age 6% and 5.3%, respectively, had certified sickness absence. The group consisting of the oldest participants (≥ 56 years of age) had a lower prevalence of certified sickness absence (3.1%) and also a lower reported level of exposure to work-related violence and/or threats of violence (7.5%).

### Logistic regression analysis

Table 2 shows odds ratios with p-values and confidence intervals from the logistic regression analyses on exposure to work-related violence and/or threats of violence and certified sickness absence due to mental disorders.

**Table 2:** Odds Ratio of certified sickness absence due to mental disorders following exposure to workplace violence and/or threats of violence. Stratified by sex

	*N*	Cases	%	Crude	Model 1^a^	Model 2^b^
				OR (95% CI)	OR (95% CI)	OR (95% CI)
All	16 339	753	5			
Exposed	1433	114	8	1.93 (1.57–2.37)*	1.59 (1.28–1.98)*	1.46 (1.17–1.82)*
Not exposed	14 267	639	4.5	1	1	1
Women	8772	588	7			
Exposed	1013	97	9	1.57 (1.25–1.97)*	1.53 (1.20–1.93)*	1.44 (1.13–1.83)*
Not exposed	7759	491	6	1	1	1
Men	7567	165	2			
Exposed	420	17	4	1.99 (1.20–3.33)*	1.85 (1.10–3.11)**	1.52 (.90–2.58)
Not exposed	7147	148	2	1	1	1
Interaction sex*exposure to violence				< 0.01	*p* = 0.46	*p* = 0.50

*p < 0.01, **p < 0.05

^a^Model 1 adjusted for age, sex, cohabitation, children living at home, socio-economic status and educational level

^b^Model 2 adjusted for age, sex, cohabitation, children living at home, socio-economic status, educational level and work-environmental factors

The OR for the crude model indicated a statistically significant association between exposure to work-related violence and/or threats of violence and certified sick absence due to mental disorders (OR 1.93, CI 1.57–2.37, *p* < *0.01*), suggesting that exposure to violence and/or threats of violence increase the odds of sickness absence due to mental disorders*.* An association was also observed when possible confounding factors such as sex, age, cohabitation, children living at home and socio-economic factors, including educational level were considered but the observed estimated odds was lower compared to the crude model (OR 1.59, CI 1.28–1.98, *p* < *0.01*) (see model 1 in Table 2).

When further adjustments were performed, where other work environment-related factors (such as factors of organizational and social work environment) were included in the model, the estimated odds were further reduced, but the result still showed a statistically significant association between exposure to work-related violence and/or threats of violence and certified sickness absence due to mental disorders. The results from the fully adjusted model indicated a 46% increase in the odds of certified sickness absence due to mental disorders after exposure to work-related violence and/or threats of violence (OR 1.46, CI 1.17–1.82, *p* < *0.01)* (see model 2 in Table 2).

Further analyses of frequency of exposure (whether exposed to violence and/or threats of violence occasionally, i.e. less than weekly, or frequent, i.e. weekly during the past 6 months) in relation to sickness absence due to mental disorders indicated a dose–response relationship (*p* = 0.001, when violence/threats of violence was treated as a continuous variable). The ORs in the fully adjusted model, however, showed no clear gradient between occasional exposure to violence and/or threats of violence (OR 1.46, CI 1.16–1.84, *p* = 0.001) and frequent exposure to violence and/or threats of violence (OR 1.44 0.80–2.60, *p* = 0.222). See Table 3 for odds ratio of certified sickness absence due to mental disorders following different frequencies of exposure to workplace violence and/or threats of violence.

**Table 3:** Odds Ratio of certified sickness absence due to mental disorders following different frequencies of exposure to workplace violence and/or threats of violence

Frequency of exposure	Crude	Model 1^a^	Model 2^b^
	OR (95% CI)	OR (95% CI)	OR (95% CI)
Occasional exposure	1.90 (1.53–3.37) *	1.59 (1.27–1.99) *	1.46 (1.16–1.84) *
Frequent exposure	2.15 (1.21–3.82) *	1.59 (.89–2.86)	1.44 (.80–2.60)

*p < 0.01, **p < 0.05

^a^Model 1 adjusted for sex, age, cohabitation, children living at home, educational level and socioeconomic status

^b^Model 2 adjusted for sex, age, civil status, children living at home, educational level, socioeconomic status, level of demands, social support and decision authority

Among the covariates, sex (being a woman: OR 3.00, 95% CI 2.50–3.60 *p* < 0.01), not having children living at home (OR 0.78, 95% CI 0.66–0.91 p < 0.05), lack of support (OR 1.26, 95% CI 1.11–1.43 *p* < 0.01) and high demands (OR 1.26, 95% CI 1.11–1.43 *p* < 0.01) were associated with certified sickness absence due to mental disorders, see model 2 in supplementary table 1. Hence, female sex, having children living at home, lack of support and high demands seems to increase the odds of sickness absence due to mental disorders.

### Interaction analysis

No statistically significant interaction between sex and the predictor variable exposure to violence and/or threats of violence could be observed (*p* = 0.50) for model 2, suggesting no clear difference among men and women. However, when results were stratified by sex, the fully adjusted model showed a statistically significant association between exposure to work-related violence and/or threats of violence and certified sickness absence among women but not among men (see Table 2). The statistically significant association between exposure to work-related violence and certified sickness absence among men was no longer observed after adjustment for confounding factors such as age, sex, cohabitation, children living at home, socio-economic status and educational level as well as other work environment factors (see model 2 in Table 2).

Further, when analyses were performed stratified by age (data not shown), there was a statistically significant association between exposure to work-related violence and/or threats of violence and certified sickness absence due to mental disorders in the fully adjusted model, only for the age-group consisting of participants of 36–55 years of age (OR 1.54, CI 1.18–2.02, *p* < 0.01). Among those < 35 years of age and those > 55 years of age, there was no statistically significant association between exposure to work-related violence and/or threats of violence and certified sickness absence (OR 1.28, CI 0.72–2.28, *p* > 0.05 and OR 1.25, CI 0.76–2.05, *p* > 0.05, respectively).

However, the analyses indicated no interaction between age and exposure to violence and/or threats of violence (*p* > 0.05).

Furthermore, there was no statistically significant interaction between exposure to violence and/or threats of violence and any of the included psychosocial work environment factors (social support, level of work demands and decision authority (*p* > 0.5, data not shown).

## Discussion

This study investigated if exposure to work-related violence and/or threats of violence increase the odds of certified sickness absence due to mental disorders up to six years following exposure. The results of the present study indicate that exposure to work-related violence and/or threats of violence is a predictor of certified sickness absence due to mental disorders.

These findings support and extend the results from earlier work. Previous studies have indicated that exposure to work-related violence and/or threats of violence is a risk factor for elevated levels of anxiety- and stress-related symptoms and exhaustion disorder (Mento et al. 2020) or depression (Rudkjoebing et al. 2021). Exposure to work-related violence and/or threats of violence have also been linked to sickness absence (Aagestad et al. 2016; Friis et al. 2018; Hoffmann et al. 2020), and a higher risk of sickness absence among employees in human service occupations compared to employees in other occupations partly due to exposure to workplace violence, has been observed (Aagestad et al. 2016; Aronsson et al. 2019; Friis et al. 2018; Niedhammer et al. 2013; Nyberg et al. 2021; Slany et al. 2014).

Our work is, however, the first study to our knowledge to investigate workplace violence in relation to diagnosis-specific sickness absence due to mental disorders. The evidence on associations between workplace bullying and violence and stress-related mental disorders has thus far been found to be limited and inconsistent (van der Molen et al. 2020). This study adds further support to indicate that exposure to workplace violence may give rise to mental health problems associated with significant functional limitations. This has also been supported in a couple of previous studies (Madsen et al. 2021; Wieclaw 2006). The study by Madsen et al. (2021) suggested that a high probability of exposure to violence increase the risk of depressive disorder and the study by Wieclaw (2006) that both potential exposure to violence and threats increase the risk of clinical diagnosis of affective or stress-related disorder. Both studies from Madsen et al (2021) and Wieclaw (2006) were representative studies, however, they used a job exposure matrix to determine exposure with a higher risk of misclassification of exposure. Rudkjoebing et al (2021) also studied self-reported exposure and depression but focused on public employees. The present study is, however, one of the few nationally representative studies on workplace violence and mental disorders assessed through sickness absence diagnosed by a physician.

Several earlier studies have also found evidence that there is an association between other types of adverse psychosocial work environment characteristics, such as high demands and low control and low support and justice, and stress-related health problems, such as different forms of anxiety- and depressive disorders, as well as levels of sickness absence (Duchaine et al. 2020; Nieuwenhuijsen et al. 2010; Slany et al. 2014; Stansfeld and Candy 2006; Sterud et al. 2021). Like other job stressors exposure to threatening and highly challenging actions such as violence and/or threats of violence, especially during an extended period without recuperation time, could potentially result in various forms of stress reactions such as an increase in blood pressure and heart frequency as well as higher rates of inflammation (Kivimäki and Steptoe 2018). In addition to a range of psychophysical changes, it has been suggested that job stressors could also lead to changes in health-related behaviors (Rugulies 2019), potentially explaining how exposure to violence and/or threats of violence can result in negative health consequences and by extension sickness absence.

Our analyses also pointed towards a possible dose–response relationship which is in line with results by Rudkjoebing et al. (2021) and Wieclaw (2006) who found that a higher level of exposure tended to be associated with higher risk of depression, diagnosed using SCAN-interviews, or prescribed antidepressants (Rudkjoebing et al. 2021) or medically certified diagnosed affective and stress related disorders (Wieclaw 2006). However, our results regarding dose–response relationship may need to be interpreted cautiously since ORs were similar for occasional and frequent exposure. The number of individuals frequently exposed to work-related violence were few, which may have limited our ability to observe differences according to frequency of exposure.

An interaction between work-related violence and psychosocial work environment factors could have indicated mechanisms or alternative intervention strategies. Workplace social support have for instance been suggested to buffer the negative consequences of exposure to bullying and harassment (Blomberg and Rosander 2020; Nielsen et al. 2020). However, the results of the present study did not show an interaction between exposure to work-related violence and/or threats of violence and psychosocial work environment factors. These results support the work by, e.g. Hoffmann et al. (2020) who did find that work-place violence and low social support independently was associated with sickness absence but, as in the current study, did not observe an interaction between the two (Hoffmann et al. 2020). The authors thus concluded that social support does not seem to buffer the effect of workplace violence on the risk of long-term sickness absence. The results of our study indicated that resources such as decision authority and low demands at work are also unlikely to buffer the association between work-related violence and sickness absence.

Together with existing literature, these results may contribute to improved training for employers and employees for prevention of ill-health, recommendations and regulations that could be implemented at workplaces. These results further emphasize the importance of conducting risk assessments focusing on the risk of exposure to violence and/or threats of violence at every workplace, and furthermore to develop action plans that include access to necessary support to be activated when needed. More knowledge seems, however, warranted on organizational factors and work environment factors contributing to occurrence of workplace violence or that could moderate the relationship between work-related and mental disorders, factors that could be targeted for prevention of workplace violence or the negative consequences of work-related violence.

## Strengths and limitations

The study was conducted as a prospective cohort study with a large and fairly representative sample of the working population in Sweden.

The cohort consisted of individuals living in different parts of Sweden, working in different occupations and with different socio-economic status and educational levels, factors that contribute to the study’s high external validity and increases the possibility to draw conclusions regarding the population as a whole. The fairly representative sample of the working population in Sweden, not focusing on any specific category of occupation, is also considered to be a major strength. However, the fact that the study does not focus on any particular sector or occupation could also be considered a limitation since it potentially lowers the possibility to use the results for preventive measures at specific workplaces.

Methodologically, the study was based on a combination of self-reported survey data and official register data of certified sickness absence. A strength with this approach is that exposure and outcome are assessed by different sources limiting common method bias, which is also further limited by the fact that the study is prospective in its design. Another strength of the present study was the use of register data to measure the outcome of certified sickness absence. To our knowledge the present study is the only published study, focusing on exposure to violence and/or threats of violence, where objective measures of mental disorders was used in form of certified sickness absence. Earlier studies on mental disorders have used clinical interviews, questionnaires or prescriptions of anti-depressive medication as a measure of outcome (Andersen et al. 2019; Magnavita 2013; Rudkjoebing et al. 2021; Sterud et al. 2021); however, as previously mentioned, self-reported measurements are associated with certain limitations such as measurement error and common method bias, and further, not all stress-related mental disorders are treated with anti-depressants, and some of the participants with such disorders might, therefore, erroneously be classified as not having such disorders. We could also follow-up all participants in the register, minimizing study attrition, which is a major strength.

Further strengths with the present study are the exclusion of participants with a history of certified sickness absence due to mental disorders, which makes it more probable that work-related violence temporally preceded the sickness absence and less probable that reverse causation is an explanation for the findings. This may, on the other hand, involve a risk for selection bias and reduce the generalizability of the sample.

A possible limitation of the study is therefore that the individuals may not be fully representative of the general working population in terms of, e.g. health. There might also be higher possibility of non-participation among individuals with poor health. Another limitation might be the fact that the results are partly based on self-reported data which could mean a risk for non-differential misclassification, as the ratings of exposure to violence may be affected by poor mental health.

The fact that exposure to violence and/or threats of violence was measured using a one-question measurement that does not distinguish exposure of physical violence from threats of violence could also be a limitation. The way exposure to certain incidents and situations is experienced and perceived could be highly influenced by individual perception and awareness, as well as the cultural and geographical context to where it takes place. Therefore, using a one-question measurement of whether the individual has been exposed to violence and/or threats of violence could mean a risk of misclassification.

Further, in the register used as measure of our outcome variable (MiDAS), only the first diagnosis in a certified sickness absence spell is registered. Some cases of sickness absence due to mental disorders could thereby have been missed. This potential misclassification is, however, likely to be non-differential leading to an underestimation of the association rather than overestimation.

No gender or age differences were found in the present study. However, the lack of differences could possibly be attributed to a lack of power. Even though the study in total included a large study sample, each separate group (for example men that have been exposed to work-related violence and/or threats of violence and have a new sickness absence spell due to common mental disorder) were fairly small which could have contributed to lack of statistical power for subgroup analyses.

An additional strength of the present study was adjustment for potential confounding variables when performing the statistical analyses. The observed association was statistically significant even after controlling for possible confounding variables, such as demographic factors, including cohabitation and children living at home, and socio-economic and demographic factors such as socio-economic status and educational level. Further, the model was adjusted for other work environment factors, such as support from supervisors and co-workers, level of demands and decision authority. However, bias due to unmeasured confounding as an explanation to the observed relationship between exposure to violence and/or threats of violence and certified sickness absence cannot be ruled out, and further studies are needed to fully map the factors behind sickness absence due to mental disorders. Such unmeasured confounding could potentially include genetic factors and childhood adversity.

## Concluding remarks

In this large nationwide prospective cohort study, exposure to work-related violence and/or threats of violence was indicated as a predictor of certified sickness absence due to common mental disorders. Thus, preventive measures as well as routines for identification of and dealing with this type of serious work-related exposure, such as exposure to violence and/or threats of violence in the work setting, is of great importance and could contribute to reduce the significant societal and economic burden associated with sickness absence due to mental disorders.

## Supplementary Information

Below is the link to the electronic supplementary material.

Supplementary file1 (DOCX 34 KB)

## Data Availability

A strategy for data access has been developed to protect personal privacy of the participants. Requests for data for specific research projects or collaborations can be addressed to [data@slosh.se].
