# Supplementary material for: Exposure to work-related violence and/or threats of violence as a predictor of certified sickness absence due to mental disorders: a prospective cohort study of 16,339 Swedish men and women in paid work
Source: Int Arch Occup Environ Health. 2022 Sep 7;96(2):225–36. doi: 10.1007/s00420-022-01917-w (PMC9905169; doi:10.1007/s00420-022-01917-w)
Supplement: Supplementary file 1 — Supplementary file1 (DOCX 34 KB) [file 420_2022_1917_MOESM1_ESM.docx]

|  |  |  |  |  |  |  |
| --- | --- | --- | --- | --- | --- | --- |
|  |  |  |  |  |  |  |
|  | Crude model | | Model 1^a^ | | Model 2^b^ | |
| Variable | OR | CI 95% | OR | CI 95% | OR | CI 95% |
| Exposure to violence and/or threats of violence | 1.93* | 1.57-2.37 | 1.59* | 1.28-1.98 | 1.46* | 1.17-1.82 |
| Age |  |  | .98* | .97-.99 | .97* | .97-.99 |
| Women |  |  | 3.0* | 2.54–3.64 | 3.00* | 2.50–3.60 |
| In marriage or cohabiting |  |  | 1.06 | .87-1.29 | 1.09 | .89-1.33 |
| Not having children living at home |  |  | .78* | .66-.92 | .78* | .66-.91 |
| Education level 1^c^ |  |  | .83 | .54-1.26 | .83 | .54-1.27 |
| Education level 2^c^ |  |  | .99 | .80-1.22 | 1.00 | .87-1.23 |
| Education level 3^c^ |  |  | 1.02 | .74-1.39 | 1.03 | .76-1.42 |
| Low socio-economic status^d^ |  |  | .93 | .68-1.26 | .90 | .66-1.24 |
| Middle low socio-economic status^d^ | |  | 1.09 | .81-1.46 | 1.07 | .79-1.44 |
| Middle high socio-economic status^d^ | |  | 1.16 | .88-1.53 | 1.18 | .89-1.55 |
| High socio-economic status^d^ |  |  | 1.0 | .82-1.23 | 1.0 | .82-1.23 |
| Support (higher value lower support) |  |  |  |  | 1.18** | 1.04-1.34 |
| Demands (higher value higher demands) |  |  |  |  | 1.26* | 1.11-1.43 |
| Decision authority (higher value lower decision authority) |  |  |  |  | 1.08 | .97-1.20 |
|  |  |  |  |  |  |  |

**Supplementary table 1**. Odds Ratio (OR) and 95% confidence interval (CI) of certified sickness absence due to mental disorders following exposure to workplace violence and/or threats of violence, and according to covariates.

^a^Model 1 adjusted for age, sex, cohabitation, children living at home, socio-economic status and educational level.

^b^Model 2 adjusted for age, sex, cohabitation, children living at home, socio-economic status and educational level and work-environmental factors.

^c^Education level 4, university ≥3 years including post graduate education was used as reference.

^d^Professionals and other non-manual employees, including upper-level executives was used as reference.

* *p*<.01, ** *p*<.05

**Journal: International Archives of Occupational and Environmental Health**

**Title:**

**Exposure to work-related violence and/or threats of violence as a predictor of certified sickness absence due to mental disorders. A prospective cohort study of 16339 Swedish men and women in paid work**.

Maria Wijkander, MSc, ^1^ Kristin Farrants, PhD, ^2^ Linda L Magnusson Hanson, PhD ^1^ *

**Affiliations:**

^1^Stress Research Institute, Department of Psychology, Stockholm University, Stockholm, Sweden.

^2^Division of Insurance Medicine, Department of Clinical Neuroscience, Karolinska Institute, Stockholm, Sweden.

*Corresponding author

**Address of correspondence:**

Linda L. Magnusson Hanson, Stress Research Institute at Department of Psychology, Stockholm University, 106 91 Stockholm, Sweden. Email: Linda.Hanson@su.se
